# Prenatal stress induces changes in PAR2- and M3-dependent regulation of colon primitive cells

**DOI:** 10.1152/ajpgi.00061.2022

**Published:** 2022-10-25

**Authors:** Mathieu Berger, Laura Guiraud, Alexia Dumas, David Sagnat, Gaëlle Payros, Corinne Rolland, Nathalie Vergnolle, Céline Deraison, Nicolas Cenac, Claire Racaud-Sultan

**Affiliations:** ^1^Institut de Recherche en Santé Digestive, INSERM U1220, Institut National de Recherche pour l’Agriculture, l’Alimentation et l’Environnement, Ecole Nationale Vétérinaire de Toulouse, University of Toulouse, Toulouse, France; ^2^Department of Physiology and Pharmacology, Cumming School of Medicine, University of Calgary, Calgary, Alberta, Canada

**Keywords:** intestinal stem cells, M3, PAR2, prenatal stress, sexual dimorphism

## Abstract

Prenatal stress is associated with a high risk of developing adult intestinal pathologies, such as irritable bowel syndrome, chronic inflammation, and cancer. Although epithelial stem cells and progenitors have been implicated in intestinal pathophysiology, how prenatal stress could impact their functions is still unknown. We have investigated the proliferative and differentiation capacities of primitive cells using epithelial crypts isolated from colons of adult male and female mice whose mothers have been stressed during late gestation. Our results show that stem cell/progenitor proliferation and differentiation in vitro are negatively impacted by prenatal stress in male progeny. This is promoted by a reinforcement of the negative proliferative/differentiation control by the protease-activated receptor 2 (PAR2) and the muscarinic receptor 3 (M3), two G protein-coupled receptors present in the crypt. Conversely, prenatal stress does not change in vitro proliferation of colon primitive cells in female progeny. Importantly, this maintenance is associated with a functional switch in the M3 negative control of colonoid growth, becoming proliferative after prenatal stress. In addition, the proliferative role of PAR2 specific to females is maintained under prenatal stress, even though PAR2-targeted stress signals Dusp6 and activated GSK3β are increased, reaching the levels of males. An epithelial serine protease could play a critical role in the activation of the survival kinase GSK3β in colonoids from prenatally stressed female progeny. Altogether, our results show that following prenatal stress, colon primitive cells cope with stress through sexually dimorphic mechanisms that could pave the way to dysregulated crypt regeneration and intestinal pathologies.

**NEW & NOTEWORTHY** Primitive cells isolated from mouse colon following prenatal stress and exposed to additional stress conditions such as in vitro culture, present sexually dimorphic mechanisms based on PAR2- and M3-dependent regulation of proliferation and differentiation. Whereas prenatal stress reinforces the physiological negative control exerted by PAR2 and M3 in crypts from males, in females, it induces a switch in M3- and PAR2-dependent regulation leading to a resistant and proliferative phenotype of progenitor.

## Graphical abstract

**Figure:**
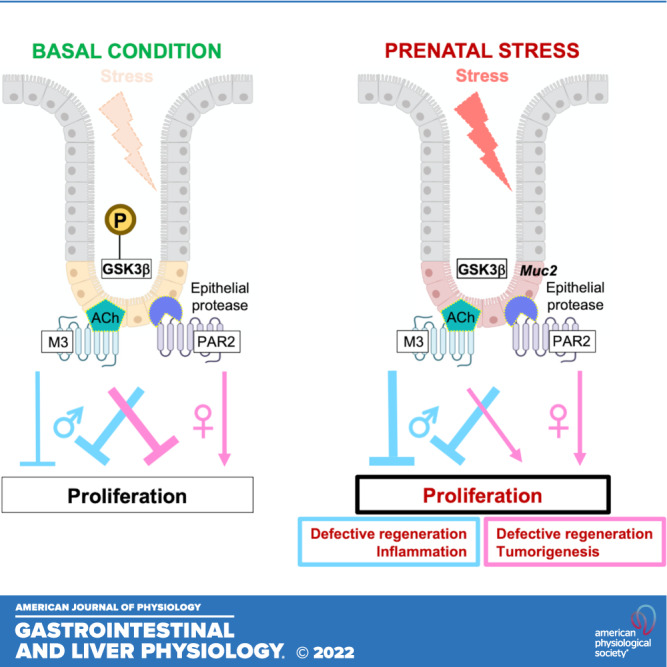

## INTRODUCTION

Prenatal stress (PS) is associated with an increased risk of a wide range of diseases during childhood and adult life (1, 2). A brain-gut axis involving neural, endocrine, and inflammatory mechanisms may be at the origin of PS-induced gut disorders such as irritable bowel syndrome (IBS), inflammatory bowel diseases (IBD, including Crohn’s disease and ulcerative colitis) and colorectal cancer (CRC) (3, 4).

Intestinal stem cells (ISCs) appear to play a central role in the pathophysiology of IBS (5), IBD (6), and CRC (7). Shah and Coll have demonstrated that gestational psychological stress alters crypt growth in a murine model (8). Even if they have shown that a corticosterone treatment depletes ISC in an intestinal explant model (8), the mechanism underlying the effects of PS on ISC requires to be elucidated.

In a recent study, we have depicted a survival/proliferative pathway depending on the protease-activated receptor 2 (PAR2) and glycogen synthase kinase 3β (GSK3β) in colon primitive cells (9). Here our aim was to study this pathway in a PS murine model. Indeed, in response to an adhesive stress, intestinal stem cells (ISCs) and colon progenitors survival is supported by the activation of GSK3β pathway downstream of PAR2 (9). Of note, this pathway is associated with a negative control of ISC/progenitors proliferation in male mice, whereas these cells remain proliferative under PAR2 activation and GSK3β inhibition in females (9). The connection between PAR2 and GSK3β appears to play a critical role in gastrointestinal disorders (IBS, IBD, CRC) since both molecules were found overexpressed/overactivated in the pathological epithelium, and conversely their inhibition improves symptoms, tissue injury and therapy (10–13). We also focused our investigation on the role of the muscarinic receptor M3 (14, 15), and hypothesized that this regulator of ISC functions could share compensatory mechanisms with PAR2 as it was previously reported in salivary glands (16).

As PS model, we have chosen stress by light and contention of rodents at the end of the pregnancy because this model, as others using psychological stress, is known to have no strong impact on pups number, sex, and weight at birth (17, 18). We used organoid culture to study colon ISC and progenitors from control and PS progenies. Indeed, organoids reflect imprinted capacities of proliferation and differentiation of ISC and their progenitors (19). Given the great impact of sexual dimorphism on the brain-gut axis (20) and ISC regulation (21), we have studied the consequences of PS on colon organoids (colonoids) from both male and female progenies.

## MATERIALS AND METHODS

### Ethics Statement

All procedures were performed in accordance with the Guide for the Care and Use of Laboratory Animals of the European Council, approved by the Animal Care and Ethics Committee of US006/CREFE (CEEA-122; application number APAFIS no. 16385-CE2018080222083660V3), and reported in accordance with the ARRIVE guidelines.

### Antibodies and Pharmacological Tools

Monoclonal antibodies: P(Ser9) GSK3β clone D85E12 (Cell Signaling Technology, Cat. No. 5558, RRID:AB_10013750, Ozyme, Saint Quentin Yvelines, France; used at 1/400); GSK3β clone 7 (BD Biosciences, Cat. No. 610202, RRID: AB_397601, Le Pont de Claix, France; used at 1/100); CD24 clone M1/69 (BD Biosciences, Cat. No. 557436, RRID: AB_396700; used at 1/100). Alexa Fluor 488/555-conjugated secondary antibodies (Invitrogen Molecular Probes, Thermo Fisher Scientific, Illkirch, France; used at 1/1,000). Pharmacological inhibitors: AEBSF [4-(2-aminoethyl) benzenesulfonyl fluoride hydrochloride], Pilocarpine and atropine from Sigma-Aldrich (Saint-Quentin Fallavier, France); 4-DAMP from Tocris Bioscience (RD Systems, Lille, France); SLIGRL-NH2 from Genscript (Piscataway, NJ); GB83 from Axon Medchem (Reston, VA).

### Animals and Prenatal Stress Model

After their purchase (Janvier Labs, Saint Quentin Fallavier, France), female and male mice (C57BL/6J, RRID: IMSR_JAX:000664) were at the zootechnic facility (ANEXPLO/Genotoul, UMS US006/INSERM, Toulouse, France) under specific pathogen-free (SPF) conditions. Our animal care facility has a SPF health status, which guarantees a better standardization of the models than in conventional facilities. Animals were maintained in ventilated cages (5 mice per cage) in a room at 20°C–24°C and relative humidity (40%–70%) with a 12 h light/dark cycle and given free access to food and water. C57BL/6J dams were randomly assigned to receive stress from *day 13* to *day 18* of gestation. The pregnant mice assigned to the stress group experienced bright light (100 watts) coupled to restraint for 30 min, 3 times a day, with at least 3 h between each stress session. On the last day, gestating mice were put in separated cages. The pups were weighed every 3 days to monitor their growth. On postnatal *day 21* to *28*, the pups were weaned from their mothers. The offspring (F1 generation) at 9 wk old, both male and female, were euthanized by cervical dislocation to be used in the colonoid experiments.

In some experiments, C57BL/6J mice deficient for PAR2 (PAR2 knockout, PAR2KO) (22) and wild-type (WT) C57BL/6J mice were used at 9 wk old (both male and female). WT and PAR2KO mice were obtained by homozygous crossings of parental animals and raised in the same zootechnic facility (ANEXPLO/Genotoul, UMS US006/INSERM).

### Colonoid Culture and Pharmacological Treatment

Colon crypts were isolated from the 2/3 ends of distal colon of F1 C57BL/6J male or female mice (*n* = 5 experiments with 3–9 mice of each sex with maternal stress or not) or WT/PAR2KO mice (*n* = 1 experiment with 3 mice of each sex). Each colon was opened longitudinally, washed in phosphate-buffered saline (PBS), and incubated in PBS with EDTA (9 mM), DTT (Dithiothreitol, 3 mM, Sigma-Aldrich), and Y-27632 (10 µM, Sigma-Aldrich) at 4°C for 75 min, under orbital shaking. After transfer in cold PBS, colons were shaken vigorously twice for 2 min to isolate crypt fragments. Crypts were counted and pelleted (43 *g*, 5 min), then resuspended in TRIzol (Invitrogen) for further transcriptomic analysis or in Matrigel for organoid culture.

Five-hundred crypt bottoms from each colon were embedded in 20 µL Matrigel (EHS sarcoma tumor matrix, growth factor reduced, phenol red free, BD Biosciences) and seeded in 8-well Lab-Tek (Thermo Fisher Scientific). Ten minutes after initiation of Matrigel polymerization at 37°C, 250 µL DMEM F12 supplemented with 100 U/mL penicillin-streptomycin, 10 mM HEPES, 2 mM Glutamax, N2 (1/100), B27 (1/50; all from Thermo Fisher Scientific), 100 ng/mL Wnt3a (RD Systems), 50 ng/mL EGF (Gibco, Thermo Fisher Scientific), 100 ng/mL noggin (Peprotech, Neuilly sur Seine, France), and 1 µg/mL R-spondin 1 (RD Systems) was added. Medium was changed every 2 days.

3D cultures (Colonoids) showed round shape structures whose size increased until the seventh day, when cultures were stopped for immunofluorescence (IF) analysis. At *day 6*, colonoids were counted manually (four quadrants of the Matrigel layer with highest colonoid density) through bright-field microscopy, and their growth was evaluated with images taken at Apotome microscope (Zeiss Axio-observer, HXP120) and imported into the ImageJ software (RRID: SCR_003070, image processing is described below).

PAR2 and M3 activation were respectively triggered by the peptide SLIGRL (100 µM, dissolved in HBSS) and pilocarpine (100 µM, dissolved in PBS). PAR2 and M3 inhibition were respectively performed using GB83 (2.5 µM, dissolved in DMSO) and 4-DAMP (10 µM, dissolved in DMSO). AEBSF (1 µM, dissolved in HBSS) and atropine (10 µM, dissolved in PBS) were added to the colonoid culture to inhibit all serine proteases and muscarinic receptors, respectively. Final concentrations have been determined by our previous (9) (SLIGRL) or preliminary (GB83, AEBSF) experiments, or according to the literature (14, 23). All pharmacological tools were added to the colonoids every 2 days from D0 of the culture. Note that our previous (9, 21) or preliminary experiments have shown that the control peptide with reversed sequence of SLIGRL and final concentration of DMSO (1 × 10^−4^%) did not modify colonoid growth/survival.

### Immunostaining

For immunocytostaining, colonoids in 8-well Lab-Tek were fixed in 2% paraformaldehyde (20 min), washed 3 times in PBS (15 min), and then permeabilized in PBS with 0.5% Triton X100 (20 min). After two washes in PBS with 100 mM glycine (20 min), blocking solution [7.7 mM NaN_3_, 1% bovine serum albumin (BSA), 0.2% Triton X100 and 0.05% Tween-20, in PBS] was added for 90 min. Antibodies directed to GSK3β or CD24 were incubated overnight at 4°C. After three washes in blocking solution (15 min), secondary antibody was incubated for 45 min. A control was made in the same conditions with the isotype control as primary antibody. The actin staining was performed by adding Alexa Fluor 647 phalloidin (Cat. No. A22287, Invitrogen, 1/40, 15 min) followed by three washes in PBS before mounting. Then after washes in PBS, slides were mounted in Prolong Gold antifade mountant with DAPI (Invitrogen) and observed by confocal laser scanning (Zeiss LSM710). Images were analyzed after their importation into the ImageJ software (image processing is described below).

Histological sections from frozen mice colons (3–4 sections per mouse from 3 control male or female mice and 3 PS male or female mice) in optimum-cutting temperature compound were prepared. Tissues were fixed with 4% formaldehyde. After three washes (3 × 10 min) in PBS plus 0.5% Triton X-100, and 1% BSA, slides were incubated overnight with primary antibodies in PBS-Triton X-100-BSA. After three washes in PBS-Triton X-100-BSA, slides were then incubated with secondary fluorescent-coupled antibodies for 2 h at room temperature. After washes in PBS, samples were mounted in Prolong Gold DAPI and observed by confocal microscopy as described above.

### Reverse Transcriptase-Polymerase Chain Reaction

Isolated crypts were conserved at −80°C in TRIzol until RNA extraction. Total RNAs from around 1 × 10^4^ crypts from each colon were extracted using the Direct-zol RNA kit (Zymo Research, Ozyme France) according to manufacturer’s instructions. Nucleic acid quantity and purity were assessed by the absorbance A_260_ and the ratio A_260_/A_280_, respectively (Nanodrop 2000, Thermo Fisher Scientific). 1 µg RNA was reverse transcribed in 20-µL reaction volume using the Maxima first strand kit and following the manufacturer’s instructions (Fermentas, Thermo Fisher Scientific). Quantitative PCR was prepared with Takyon NO ROX SYBR Mmx dTTP blue (Eurogentec, Belgium) and 45 ng cDNA was used as template for amplification (40 cycles, 60°C) using 0.6 µM gene-targeted primers (Table 1). The run was performed in two technical replicates on a LightCycler 480 Instrument (Roche). All primers used have PCR efficiency >90%. *Hprt* and *Gapdh* were used as reference genes. The minus delta Ct was calculated (Microsoft Excel software, RRID: SCR_016137) from housekeeping gene (*Hprt*, *Gapdh*) to target gene duplicates. Comparative data shown were calculated from DdCt with *Hprt* as reference gene (similar data were obtained with *Gapdh* as reference gene).

**Table 1.** Oligonucleotides used for quantitative RT-PCR

Genes	NCBI Accession Number	5′ Forward 3′ 5′ Reverse 3′
*Dusp6*	NM_026268.3	CAAGCAAATTCCTATCTCGGGTCGTAAGCATCGTTCATG
*Ets2*	NM_011809	AGATGCTGTGTAACCTCGGCCTAATGTATTGCTGTTGATC
*Gsk3b*	NM_019827.7NM_001347232.1	TGGTGCTGGACTATGTTCGTTCTGTGGTTTAATGTCTCG
*Lgr5*	NM_010195	CTACTCGAAGACTTACCCAGTGCATTGGGGTGAATGATAG
*Lrig1*	NM_008377	ACAATCGAGGATACCAGTGTCCAAGGTTCAGGTGTTC
*Sox9*	NM_011448	GAGCCGGATCTGAAGAGGGAGCTTGACGTGTGGCTTGTTC
*Bmi1*	NM_007552	TCCCCACTTAATGTGTGTCCTCTTGCTGGTCTCCAAGTAACG
*Snai1*	NM_011427	GCGGAGTTGACTACCGACCGAAGGTGAACTCCACACACG
*Wnt5a*	NM_001256224NM_009524	GTCCTTTGAGATGGGTGGTATCACCTCTGGGTTAGGGAGTGTCT
*Ngn3*	NM_009719	GTCGGGAGAACTAGGATGGCGGAGCAGTCCCTAGGTATG
*Muc2*	NM_023566	GTAAACTGCTCTCTGGACTGCTTGGAAGACGTGGTAGATG
*Dclk1*	NM_019978	CTGCAGCAGGAGTTTCTGTACCGAGTTCAATTCCGGTGGA
*Chga*	NM_007693	TCCCCACTGCAGCATCCAGTTCCCTTCAGACGGCAGAGCTTCGG
*Slc26a3*	NM_021353	AGCCGATCAATACCACAGCCGAGCAAGTTTGTCAATG
*Hnf1*	NM_009327	AAAACCCCAGCAAGGAAGAGGGTTGGCAAACCAGTTGTAG
*Atoh1*	NM_007500	GCTTATCCCCTTCGTTGAATCTTTTACCTCAGCCCAC
*Klf4*	NM_010637	GACTAACCGTTGGCGTGAGGGTCTAGGTCCAGGAGGTCGT
*F2r* (PAR1)	NM_010169.4	TCTTCCCGCGTCCCTATGAGGGGTTCACCGTAGCATCTG
*F2rl1* (PAR2)	NM_007974.4	GGACCGAGAACCTTGCACGAACCCCTTTCCCAGTGATT
*F2rl3* (PAR4)	NM_010170	AGCTGAGGGGAATCTACGCTAGGTTGGCTTTGCTGAGTTG
*Cux1*	NM_001291233NM_001291234NM_009986	TCCGTAGCATCCAAGGCAGACACTTCATCAGAACCAGTCTCAGA
*Gna15*	NM_010304	GTGATTGCCCTCATCTACCTGGATGACCGAGGTGCTCTTGAACC
*Mapk3*	NM_011952.2	CACTGGCTTTCTGACGGAGTGGATTTGGTGTAGCCCTTGGA
*Timp2*	NM_011594.3	CAACAGGCGTTTTGCAATGCATCCTCTTGATGGGGTTGCC
*Itga2*	NM_008396	ACCCACGGAGAAAGCAGAAGCGCCGATGGTTTAGCTGTTG
*Itga3*	NM_013565.3NM_001306162.1	GATTCCTGGTGGTGAAGGAGGGGGACACAGGTACACAGCAC
*Itga6*	NM_008397.4NM_001277970.1	CTCCCTCTCAGACTCGGTCACTGGCGGAGGTCAATTCTGT
*Itgb4*	NM_133663NM_001005608	ACTGCAAGGAGAACGCATCTCAGACTCGGTGGAGAACACC
*Chrm1*	NM_007698NM_001112697	AGCAGCAGCTCAGAGAGGTCGCCTGTGCCTCAGGATCTAC
*Chrm3*	NM_033269	TGGTGTGTTCTTCCTTGGACACCCAGGAAGAGCTGATGTT
*Ache*	NM_001290010NM_009599	CCTGGGTTTGAGGGTACTGAGGTTCCCACTCGGTAGTTCA
*Buche*	NM_009738	GGGCAGTAAAGCATCCTGAGGAGGGGAGAACGAACCTTTC
*Prox1*	NM_008937NM_001360827	GCTATACCGAGCCCTCAACAATCCAGCTTGCAGATGACCT
*Trpv4*	NM_022017	TCACCTTCGTGCTCCTGTTGAGATGTGCTTGCTCTCTCCTTG
*Hprt1*	NM_013556	TCAGTCAACGGGGGACATAAAGGGGCTGTACTGCTTAACCAG
*Gapdh*	NM_001289726	AGGTCGGTGTGAACGGATTTGTGTAGACCATGTAGTTGAGGTCA

Official gene symbols, NCBI accession number of targeted transcripts, and forward and reverse oligonucleotide sequences are depicted.

### Image Processing and Statistical Analysis

Apotome and confocal images were imported into the ImageJ software for analysis. Size (diameter) of around 20 colonoids was measured in each assay. A threshold ≥45 µm (Crypt bottom diameter) was taken for the study of colonoid growth. IF quantification was performed after image binarization and measures were reported to the mean fluorescence in control assays.

For each experiment (animals, crypts, colonoids), male and female were processed simultaneously. Statistical analyses were performed using GraphPad Prism 9 software (RRID: SCR_002798). Student’s *t* test was used for experiments analysis. *P* values <0.05 were considered to be significant.

## RESULTS

### Balanced Functions of PAR2 and M3 in Colon Primitive Cells

In regard to a role of PAR2 and M3 on growth control of colon primitive cells (9, 14, 15), we first investigated their potential crosstalk in colonoids cultured into basal conditions, in presence of various pharmacological drugs (GB83 and 4-DAMP, antagonists of PAR2 and M3, respectively; SLIGRL and Pilocarpine, agonists of PAR2 and M3, respectively), distinguishing their functions between both males and females.

**Figure 1.**
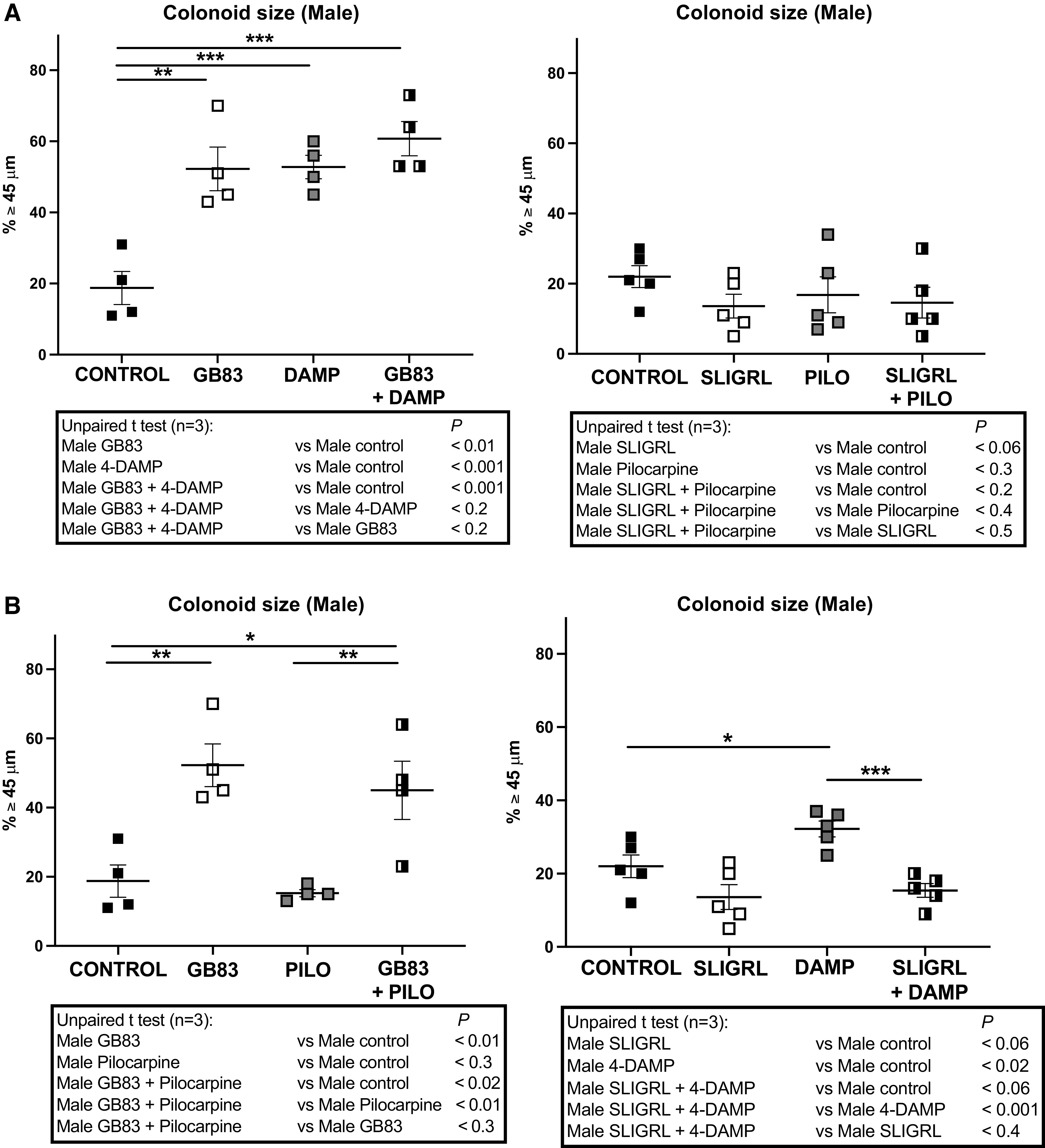
Impact of PAR2 and M3 modulation on colonoids growth from male mice. Colon crypts were isolated from male mice and cultured as colonoids. *A* and *B*: pharmacological antagonists or agonists of PAR2 and M3 were added at the beginning of the culture and every 2 days when medium was changed. Colonoids growth was evaluated at *day 6* of culture through diameter measurement of circa 20 colonoids per assay, and percentage of colonoids above the 45-µm threshold (crypt diameter) is represented (means ± SE). Data are from 3 independent experiments (*n*) with 1–2 animals per condition. The statistical *t* test is shown: *P < 0.05; **P < 0.01; ***P < 0.001. PAR2 and M3 antagonists: GB83 2.5 µM, 4-DAMP 10 µM, respectively; PAR2 and M3 agonists: SLIGRL 100 µM, Pilocarpine 100 µM, respectively.

As shown in Fig. 1*A* (*left*), the treatment by an antagonist of PAR2 or of M3 increased the size of colonoids harvested from naive (control) male mice. However, the treatment with an agonist of PAR2 or of M3 did not significantly change the colonoid size (Fig. 1*A*, *right*), showing that the negative control of organoid growth by both receptors is already very active in our culture conditions. There was no synergic effect after the treatment combining both antagonists (Fig. 1*A*, *left*) or both agonists (Fig. 1*A*, *right*), suggesting that both receptors act through the same signaling pathway in colon primitive cells from control male mice. However, when an antagonist of one of both receptors was combined with an agonist of the other receptor, the effect resulting corresponded to the effect observed with PAR2 antagonist (Fig. 1*B*, *left*) or PAR2 agonist (Fig. 1*B*, *right*) alone. Taken together, these data indicate that in colonoids issued from control male mice, both PAR2 and M3 negatively control colon primitive cell growth and that PAR2 seems to be more potent than M3.

**Figure 2.**
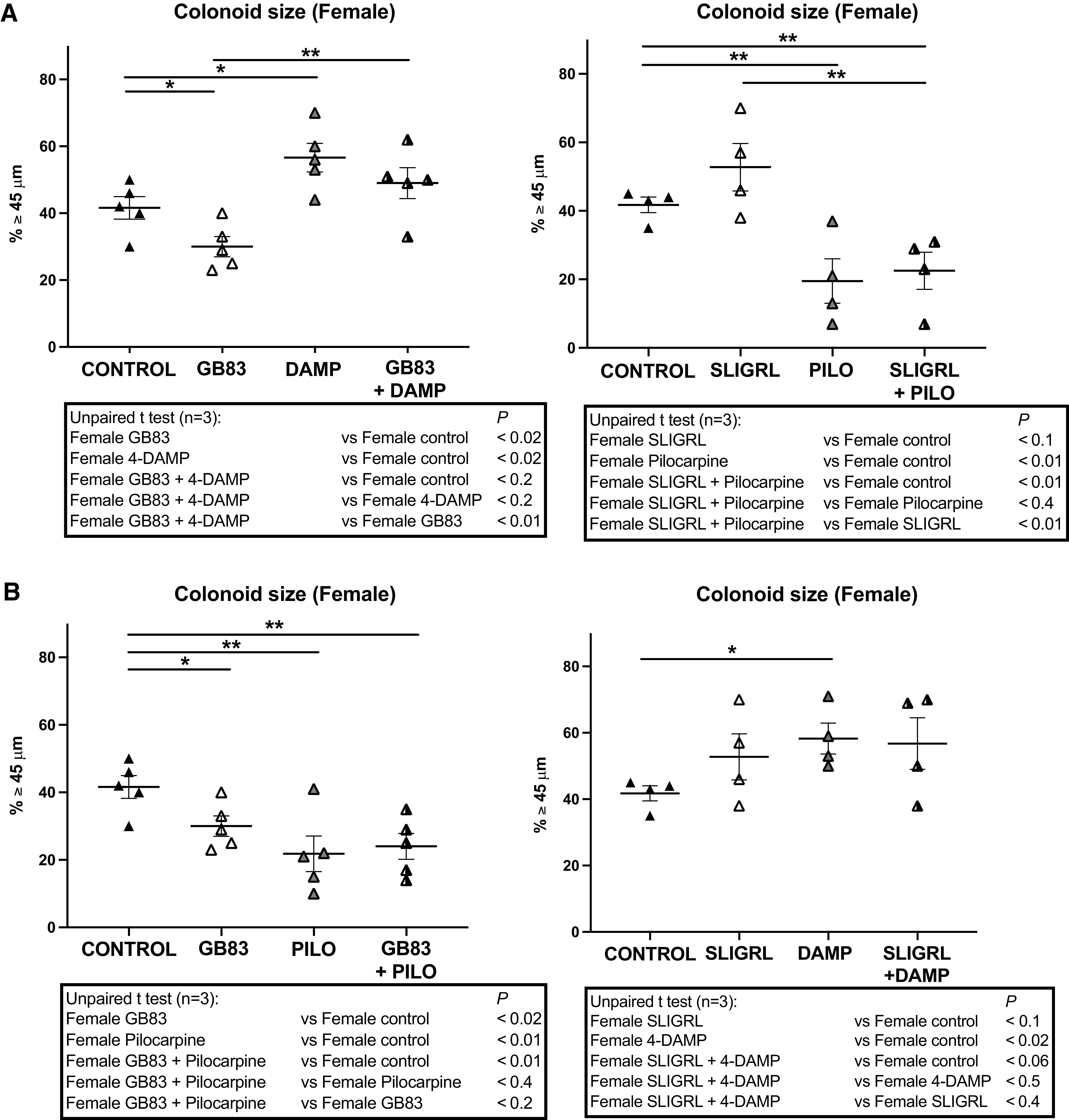
Impact of PAR2 and M3 modulation on colonoids growth from female mice. Colon crypts were isolated from female mice and cultured as colonoids. *A* and *B*: pharmacological antagonists or agonists of PAR2 and M3 were added in culture media as described in Fig. 1. Colonoids growth was evaluated at *day 6* of culture through diameter measurement of circa 20 colonoids per assay, and percentage of colonoids above the 45-µm threshold (crypt diameter) is represented (means ± SE). Data are from 3 independent experiments (*n*) with 1–2 animals per condition. The statistical *t* test is shown: *P < 0.05; **P < 0.01. PAR2 and M3 antagonists: GB83 2.5 µM, 4-DAMP 10 µM, respectively; PAR2 and M3 agonists: SLIGRL 100 µM, Pilocarpine 100 µM, respectively.

Similar experiments have been conducted on colonoids issued from control female mice. Although the treatment with an antagonist of PAR2 decreased colonoid growth, M3 antagonist increased the size of colonoids originating from control female mice (Fig. 2*A*, *left*). Accordingly, M3 agonist decreased colonoid growth, whereas PAR2 activation tended to increase size (Fig. 2*A*, *right*). Treatment combining both antagonists (Fig. 2*A*, *left*) or agonists (Fig. 2*A*, *right*) highlighted that PAR2 inhibition or activation could not counteract the effect induced by M3 inhibition or activation respectively. When targeting of both receptors induces a growth decrease (Fig. 2*B*, *left*) or increase (Fig. 2*B*, *right*), no additive or synergic effect was observed, suggesting again that PAR2 and M3 could share the same signaling pathway. Thus, in colonoids issued from control female mice, M3 negatively controls colon primitive cell growth, contrary to a positive regulation by PAR2; M3 being more potent than PAR2.

In conclusion, although M3 decreased colonoid growth from both male and female control mice, PAR2 has an opposite effect, reducing in male or improving in female. Our results strongly suggest that PAR2 and M3 are key negative regulators of growth in males and females, respectively.

### Impact of PS on Colon Primitive Cells

**Figure 3.**
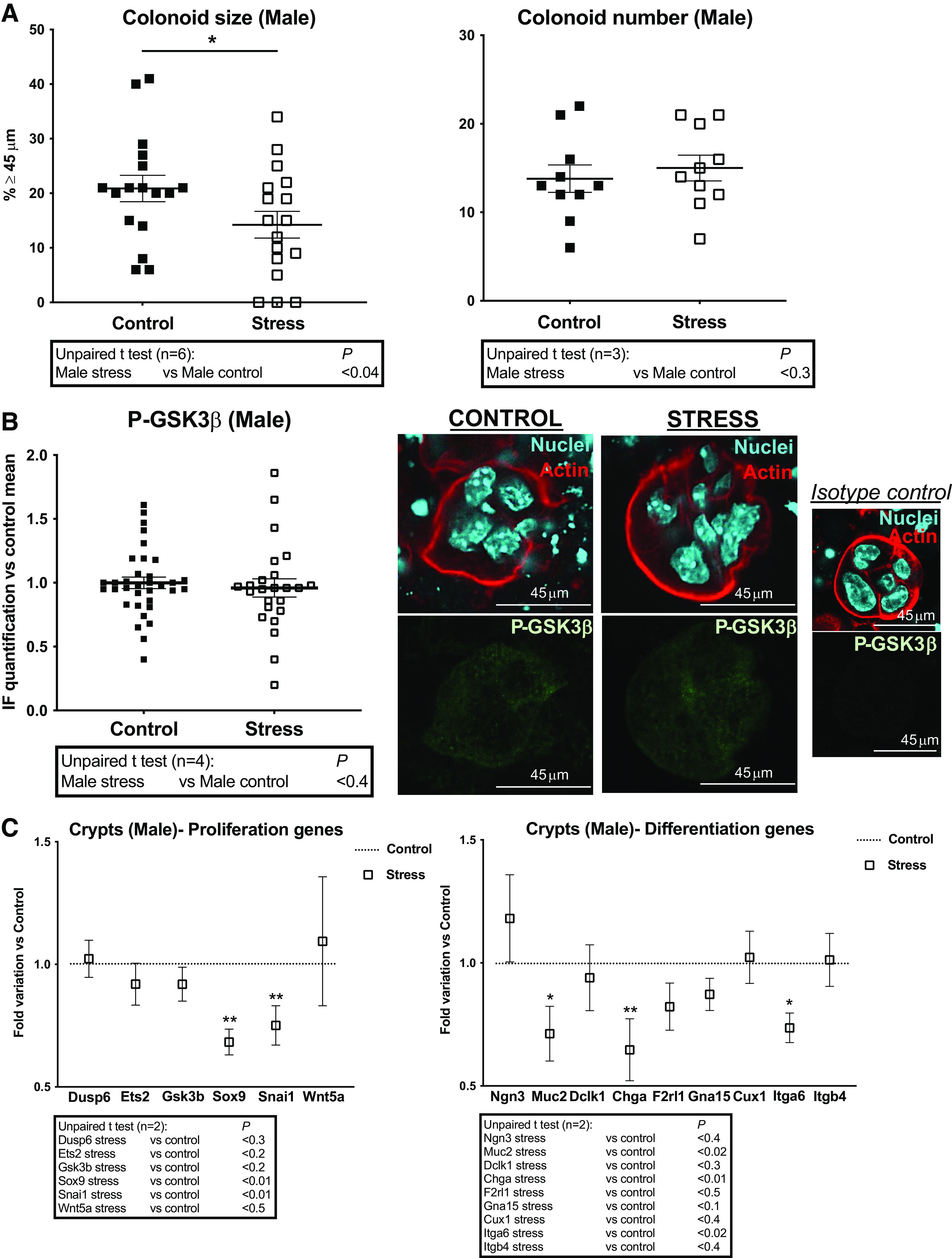
Impact of PS on proliferation and differentiation of colon primitive cells from male mice. Male mice whose mothers were stressed (Stress) or not (Control) during late gestation were used to isolate colon crypts for gene expression analysis or cultured as colonoids. *A*: colonoid growth was evaluated at *day 6* of culture through diameter measurement of circa 20 colonoids per assay, and percentage of colonoids above the 45-µm threshold (crypt diameter) is represented (means ± SE). The impact of PS on colonoid number is also shown. Data are from 3 to 6 independent experiments (*n*) with 2–3 animals per condition. *B*: immunolabeling of P-ser9 GSK3β (P-GSK3β, inhibited form) was realized on colonoids. IF was quantified from 3 to 10 colonoids per assay (4 independent experiments with 2–3 animals per condition are represented) using ImageJ software. Representation of data vs. control mean of fluorescence as means ± SE. Representative images of immunolabeled GSK3β in colonoids: actin and nuclei labeling and isotype control labeling are shown. Scale bar = 45 µm. *C*: RT-qPCR was performed on mRNA isolated from colon crypts. Expression of selected genes implicated in proliferation and differentiation of colon primitive cells is shown. Others are shown in Supplemental Fig. S1. Data (2 independent experiments with 5–7 animals per condition) are represented as fold variation vs. control. Statistical *t* tests are shown: *P < 0.05; **P < 0.01. IF, immunofluorescence; PS, prenatal stress.

Then we investigated the consequences of maternal stress (PS) on physiological properties of colon primitive cells cultured from descendance. Colonoid growth from PS (Stress) and control male mice was compared. As shown in Fig. 3*A*, colonoid size (*left*) was decreased in PS conditions whereas colonoid number (*right*) remained unchanged. These results show that proliferative capacities of ISC and progenitors are impaired by PS in male mice.

GSK3β is a stress metabolic kinase regulating stem cell/progenitor survival and proliferation (24), which negatively controls the secretory cell differentiation (Tuft and goblet cells) in the crypt, thus contributing to chronic epithelial inflammation (25). The kinase is inhibited in basal conditions and its activation under stress requires serine 9-dephosphorylation (24). Using an antibody targeting the inhibited phosphorylated form of GSK3β (P-Ser9 GSK3β), we have quantified the activated state of GSK3β in colonoids from male mice. According to our previous data (9), GSK3β was found in its active nonphosphorylated form in control colonoids obtained after stressing deadhesion of crypts from their microenvironment and cultured in conditions where cell differentiation is braked (Fig. 3*B*). Under PS (Stress), GSK3β phosphorylation state remained unchanged compared with control group (Fig. 3*B*).

The PS-induced alterations of growth in colon primitive cells prompted us to investigate gene expression of several important actors in crypts from control and PS male mice. First, the stress epicenters *Dusp6* and *Ets2* (26–28), both negative regulators of crypt primitive cell proliferation, with proinflammatory and tumor suppressor functions, were unchanged under PS, as well as *Gsk3b* (Fig. 3*C*, *left*). Whereas the expression of stem cell/progenitor markers *Lgr5*, *Lrig1* and *Bmi1* was unchanged under PS (Stress; Supplemental Fig. S1*A*, *top*), the transcription factors *Sox9* and *Snai1* target genes of growth factors in colon primitive cells (29–32) were downregulated (Fig. 3*C*, *left*), accordingly with the lower colonoid growth from PS males compared with control.

Due to the roles of PAR2 and GSK3β in the control of proliferation and differentiation of colon primitive cells (9, 21, 25), we have investigated the gene expression of a panel of factors implicated in these functions and potentially linked to a PAR2-dependent regulation. Under PS (Stress), the expression of *Muc2* and *Chga* (but not *Ngn3* and *Dclk1*) was downregulated, arguing in favor of a reinforcement of the GSK3β-dependent anti-secretory pathway (Fig. 3*C*, *right*). Also, the decrease of integrin *Itga6* (Fig. 3*C*, *right*) strongly corroborates our previous results showing a negative regulation of *Itga6* by PAR2 in males (21). The expression of PAR2 (*F2rl1*) and its other targets [*Gna15*, *Cux1*, *Itgb4*, *Itga3*, *Mapk3*, *Timp2* (21, 33, 34)], as well as other PARs (*F2r*, *F2rl3*) and differentiation markers (*Slc26a3*, *Hnf1*, *Atoh1*, *Klf4*, *Itga2*) remained unchanged under PS in males (Fig. 3*C*, *right*; Supplemental Fig. S1*A*, *top*).

**Figure 4.**
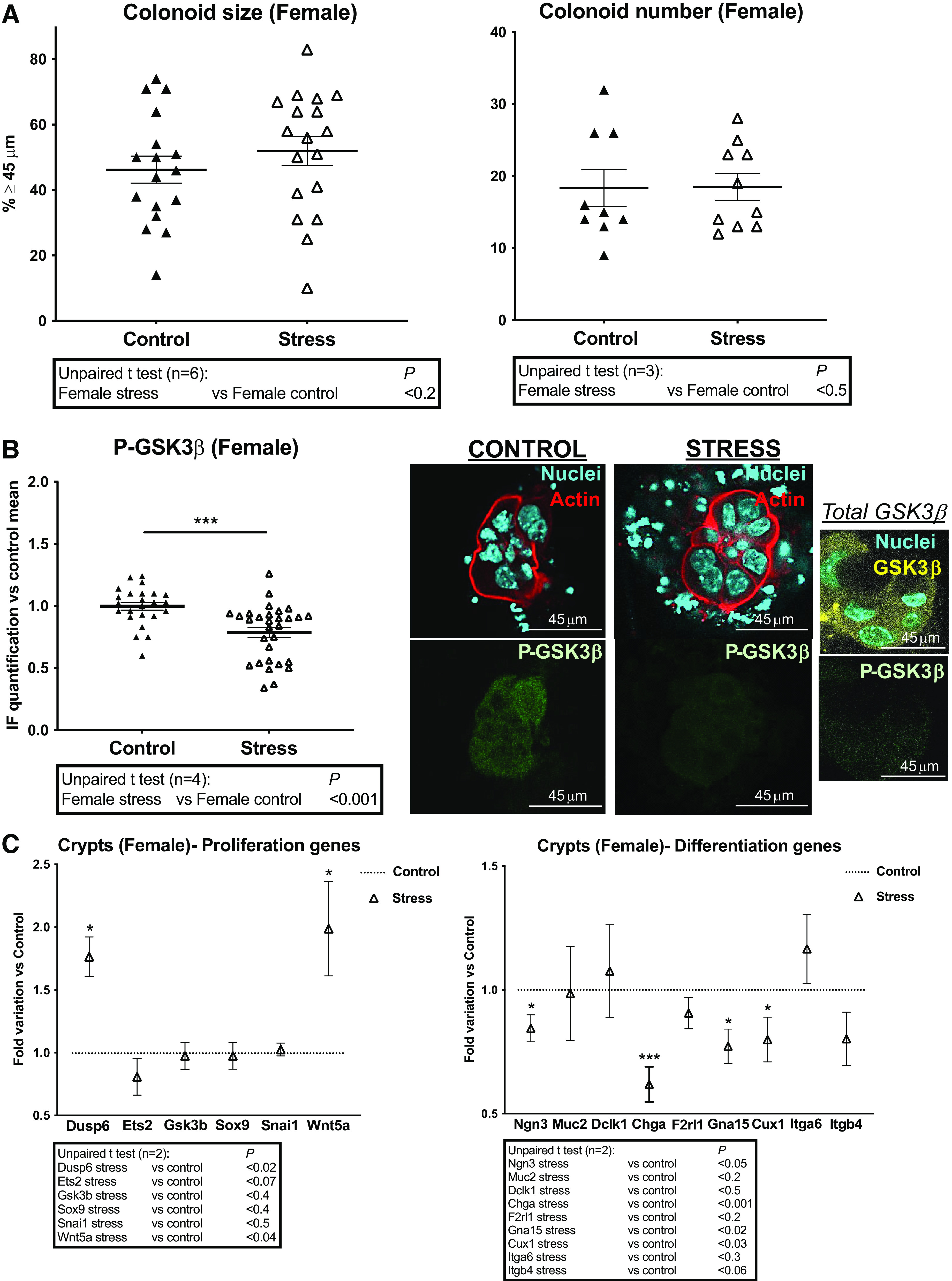
Impact of PS on proliferation and differentiation of colon primitive cells from female mice. Female mice whose mothers were stressed (Stress) or not (Control) during late gestation were used to isolate colon crypts for gene expression analysis or cultured as colonoids. *A*: colonoid growth was evaluated at *day 6* of culture through diameter measurement of *circa* 20 colonoids per assay, and percentage of colonoids above the 45-µm threshold (crypt diameter) is represented (means ± SE). The impact of PS on colonoid number is also shown. Data are from 3 to 6 independent experiments (*n*) with 2–3 animals per condition. *B*: immunolabeling of P-ser9 GSK3β (P-GSK3β, inhibited form) was realized on colonoids. IF was quantified from 4 to 10 colonoids per assay (4 independent experiments with 2–3 animals per condition are represented) using ImageJ software. Representation of data is vs. control mean of fluorescence as means ± SE. Representative images of immunolabeled GSK3β in colonoids: actin and nuclei labeling are shown. A representative image of total GSK3β *plus* nuclei labeling in PS (Stress) colonoids is shown. Scale bar = 45 µm. *C*: RT-qPCR was performed on mRNA isolated from colon crypts. Expression of selected genes implicated in proliferation and differentiation of colon primitive cells is shown. Others are shown in Supplemental Fig. S1. Data (2 independent experiments with 6–8 animals per condition) are represented as fold variation vs. control. Statistical *t* tests are shown: *P < 0.05; ***P < 0.001. IF, immunofluorescence; PS, prenatal stress.

If we compare both males and females, the size of control colonoids from females was higher than males (Figs. 1, 2, 3*A*, and 4*A*), as previously shown (21). However, colonoids size from females was unchanged in PS conditions (Fig. 4*A*, *left*) contrary to males. PS conditions did not affect the colonoid number in females (Fig. 4*A*, *right*) as well as in males. Also, we have previously shown (21) that the inhibited form of GSK3β (P-Ser9 GSK3β) predominates in colonoids from females compared with males. However, on PS (Stress), a decrease of the inhibited form of GSK3β (P-GSK3β) was measured compared with control group, despite a total expression of GSK3β maintained (Fig. 4*B*). These results show that PS modifies GSK3β regulation in colon primitive cells from female mice.

Compared with crypts from control male mice, the stress epicenters *Dusp6* and *Ets2* were respectively lower and higher expressed in control females whereas *Gsk3b* expression was similar in both sexes (Supplemental Fig. S1*B*, *top*). However, under PS (Stress), only *Dusp6* was increased in crypts from females (Fig. 4*C*, *left*) reaching the level measured in males (Supplemental Fig. S1*B*, *bottom*). Importantly, it has been demonstrated that *DUSP6* is a hub gene in females coping with stress (35). Under PS (Stress), increase of the ligand *Wnt5a* and unchanged *Sox9* and *Snai1* (Fig. 4*C*, *left*) should be related to the maintain of colonoid growth in females, given the regenerative role of Wnt5a in intestinal injury models (36).

Whereas markers of secretory cells such as *Ngn3, Muc2*, and *Dclk1*, and the enterocyte marker *Slc26a3*, were found higher expressed in females compared with males control crypts (Supplemental Fig. S1*B*, *top*), only *Ngn3* and *Chga* from the secretory pathway were decreased under PS (Stress) in females (Fig. 4*C*, *right*; Supplemental Fig. S1*A*, *bottom*). In contrast with males, *Muc2* expression was spared in crypts from PS females and as a result remained as well as *Dclk1* higher compared with PS males (Supplemental Fig. S1*B*, *bottom*). PAR2 (*F2rl1*) and other PARs (*F2r*, *F2rl3*) or PAR2-regulated targets (*Itga6*, *Itgb4*, *Itga3*, *Mapk3*, *Timp2*) were not differentially expressed between males and females, except the Gq subunit *Gna15* and the transcription factor *Cux1* (Supplemental Fig. S1*B*, *top*) that varied also specifically under PS (Stress) in females (Fig. 4*C*, *right*; Supplemental Fig. S1*A*, *bottom*).

Altogether, these results highlight PS-induced crucial and different changes in growth and differentiation of colon primitive cells, in males and females.

### Impact of PS on PAR2-Dependent Regulation of Colon Primitive Cells

**Figure 5.**
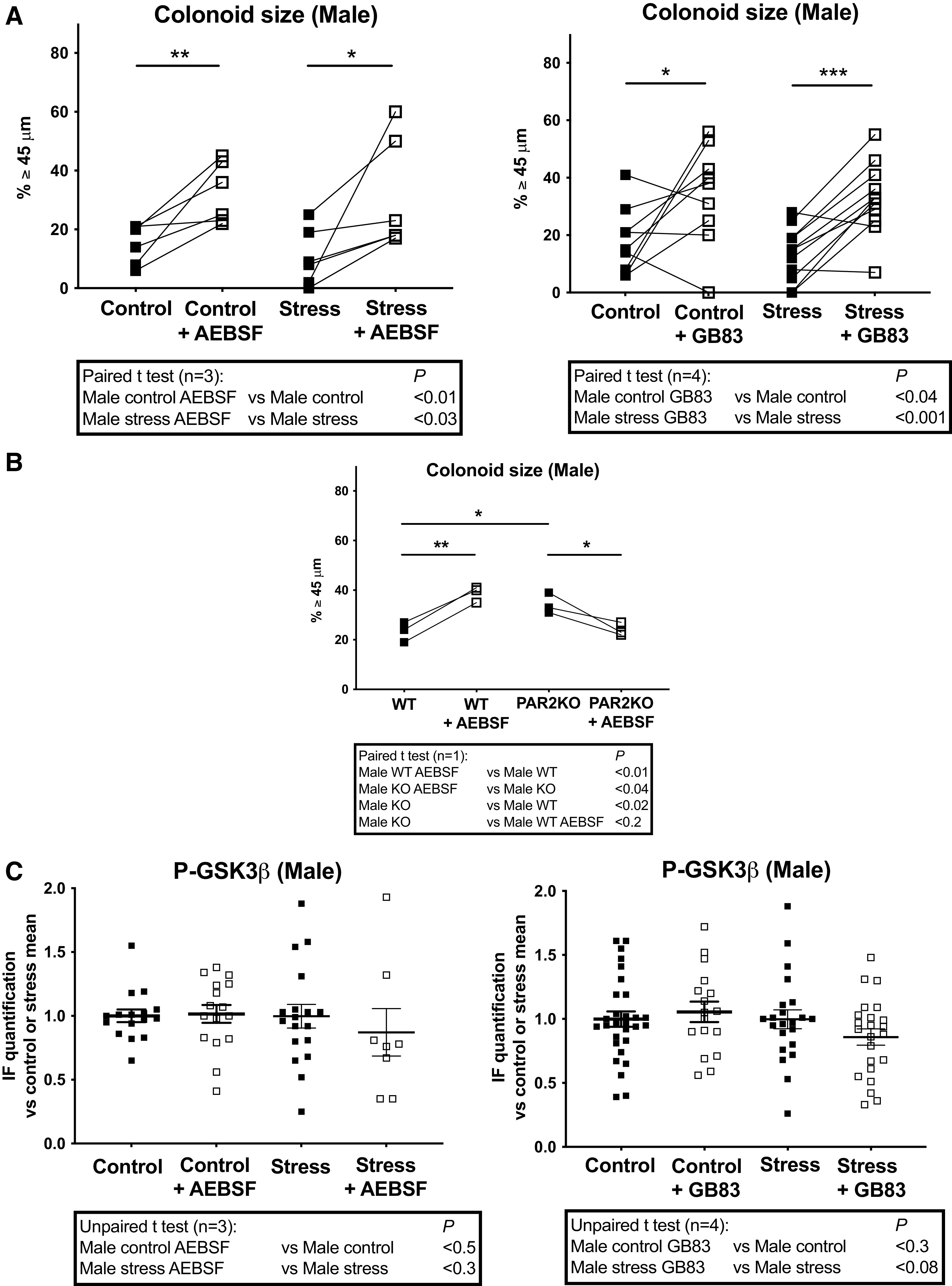
Impact of PS on PAR2-dependent regulation of colon primitive cells from male mice. Male mice whose mothers were stressed (Stress) or not (Control) during late gestation were used to isolate colon crypts that were cultured as colonoids. Serine protease and PAR2 inhibition were obtained respectively by the addition of AEBSF (1 µM) and GB83 (2.5 µM) to the colonoid culture as described in Fig. 1. *A*: colonoid growth was evaluated at *day 6* of culture through diameter measurement of around 20 colonoids per assay, and percentage of colonoids above the 45-µm threshold (crypt diameter) is represented. Lines between individual values show variations secondary to the treatment by AEBSF or GB83. *B*: colonoids from 3 WT or PAR2KO male mice were treated by AEBSF as described above. *C*: IF of P-ser9 GSK3β (P-GSK3β, inhibited form) from 5 to 10 colonoids per assay was quantified using ImageJ software. Data are from 1 to 4 independent experiments (*n*) with 1–3 animals per condition. Statistical *t* tests are shown: *P < 0.05; **P < 0.01; ***P < 0.001. IF, immunofluorescence; PS, prenatal stress; WT, wild type.

To investigate the role of PAR2 under PS, we have first measured the impact of the serine protease inhibitor AEBSF on organoid culture. Indeed, PAR2 is a receptor predominantly activated following cleavage by serine proteases (10). In males, AEBSF treatment increased colonoid size in both control and PS (Stress) conditions (Fig. 5*A*, *left*), showing that epithelial serine protease(s) are implicated in the regulation of colonoid growth. According to our previous work (21), modulation of PAR2 (here by the pharmacological inhibitor GB83) increased colonoid size in control males (Fig. 5*A*, *right*). Under PS (Stress), a more regular increase of colonoid size after PAR2 inhibition was observed (Fig. 5*A*, *right*), suggesting a critical role of PAR2 in proliferative brake and resistance facing PS in males. Importantly, in conditions where AEBSF or deletion of PAR2 (PAR2KO) triggered an increase in colonoid size, by contrast the application of AEBSF on PAR2KO colonoids induced a decrease of colonoid size (Fig. 5*B*). This demonstrates the key role of a serine protease acting through PAR2 activation in the negative control of colon primitive cells proliferation. To evaluate whether the serine protease/PAR2 pathway regulates GSK3β activity in control and PS conditions, we incubated colonoids with AEBSF or GB83 and measured the impact on its activation state. Overall measurement of P-GSK3β in colonoids treated by AEBSF or GB83 did not show significant changes in both control and PS (Stress) conditions (Fig. 5*C*).

**Figure 6.**
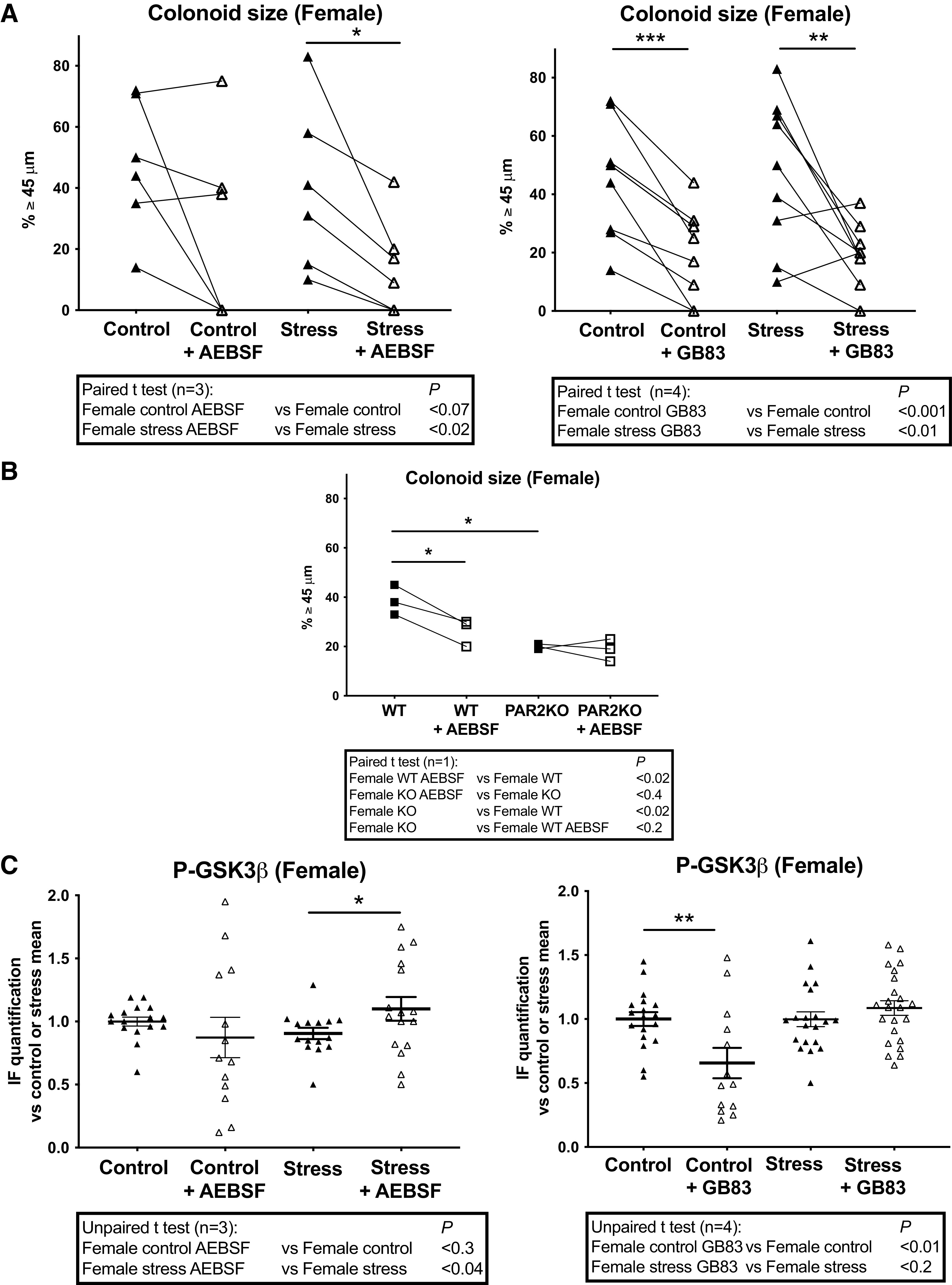
Impact of PS on PAR2-dependent regulation of colon primitive cells from female mice. Female mice whose mothers were stressed (Stress) or not (Control) during late gestation were used to isolate colon crypts that were cultured as colonoids. Serine protease and PAR2 inhibition were obtained respectively by the addition of AEBSF (1 µM) and GB83 (2.5 µM) to the colonoid culture as described in Fig. 1. *A*: colonoid growth was evaluated at *day 6* of culture through diameter measurement of around 20 colonoids per assay, and percentage of colonoids above the 45-µm threshold (crypt diameter) is represented. Lines between individual values show variations secondary to the treatment by AEBSF or GB83. *B*: colonoids from 3 WT or PAR2KO female mice were treated by AEBSF as described above. *C*: IF of P-ser9 GSK3β (P-GSK3β, inhibited form) was quantified from 5 to 9 colonoids per assay using ImageJ software. Data are from 1 to 4 independent experiments (n) with 1–3 animals per condition. Statistical *t* tests are shown: *P < 0.05; **P < 0.01; ***P < 0.001. IF, immunofluorescence; PS, prenatal stress; WT, wild type.

In contrast with males, both AEBSF and GB83 treatments decreased size of colonoids from control and PS (Stress) females (Fig. 6*A*). In conditions where AEBSF or deletion of PAR2 (PAR2KO) triggered a decrease of colonoid size, the application of AEBSF on PAR2KO colonoids did not modify colonoid size (Fig. 6*B*). This demonstrates again the key role of a serine protease acting through PAR2 activation to control colon primitive cells proliferation, here positively in females. AEBSF increased P-GSK3β in colonoids from PS (Stress) females (Fig. 6*C*, *left*) and GB83 decreased it only in colonoids from control females (Fig. 6*C*, *right*). These data strongly suggest that the serine protease/PAR2 pathway controls the GSK3β activation under PS in females.

Altogether these results confirm our previous data showing a sexual dimorphism in PAR2-dependent regulation of growth and GSK3β in colon primitive cells (21) and that an epithelial serine protease is implicated. Furthermore, under PS (Stress) the PAR2-GSK3β pathway is reinforced in males to cope with stress through proliferative and differentiation brake, whereas in females this pathway is de novo activated inducing lower differentiation capacities associated with a maintain of progenitor proliferation.

### Impact of PS on M3-Dependent Regulation of Colon Primitive Cells

**Figure 7.**
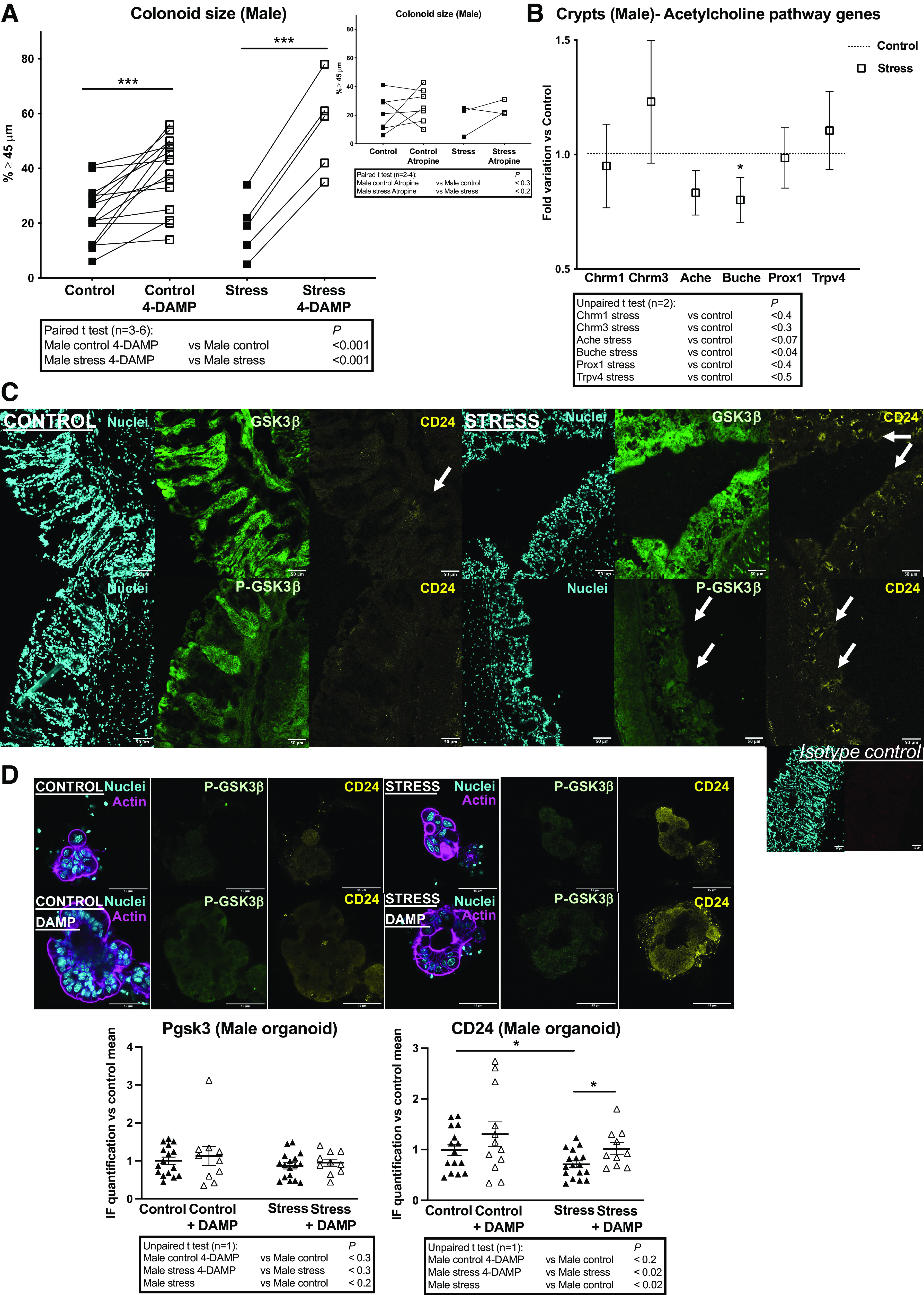
Impact of PS on M3-dependent regulation of colon primitive cells from male mice. Colon crypts were isolated from male mice whose mothers were stressed (Stress) or not (Control) during late gestation. Then colon crypts were used for gene expression analysis or cultured as colonoids. *A*: the M3 specific inhibitor 4-DAMP (10 µM) or the general muscarinic receptor inhibitor atropine (10 µM) were added to the colonoid culture as described in Fig. 1. Colonoid growth was evaluated at *day 6* of culture through diameter measurement. Around 20 colonoids were measured by assay and percentage of colonoids above the 45-µm threshold (crypt diameter) is represented. Lines between individual values show variations secondary to the treatment by 4-DAMP or atropine. Data are from 2 to 6 independent experiments (*n*) with 1–3 animals per condition. *B*: RT-qPCR was performed on mRNA isolated from colon crypts. Selected genes implicated in cholinergic regulation of colon primitive cells are shown. Data (2 independent experiments with 5–7 animals per condition) are represented as fold variation vs. Control. *C*: in situ immunolabeling of total GSK3β (green), inhibited form P-ser9 GSK3β (P-GSK3β, green) and CD24 (yellow), was realized on colon samples from male mice. Representative confocal photomicrographs of colonic tissue slices from 3 Control and 3 Stress (PS) mice (*n* = 1 experiment) are shown. Nuclei labeling (cyan) and isotype control (for CD24 antibody) labeling are shown. Scale bar = 50 µm. *D*: colonoids from Control and PS (Stress) males were treated or not with the M3 specific inhibitor 4-DAMP (10 µM) as above and immunolabeling of P-ser9 GSK3β (P-GSK3β, inhibited form) and CD24 was realized. IF was quantified from 2 to 8 colonoids per assay (1 experiment with 3 animals per condition is represented) using ImageJ software. Representation of data is vs. control mean of fluorescence as means ± SE. Representative images of immunolabeled GSK3β and CD24 in colonoids: actin (magenta) and nuclei (cyan) labeling are shown. Scale bar = 45 µm. Statistical *t* tests are shown: *P < 0.05; ***P < 0.001. IF, immunofluorescence; PS, prenatal stress.

Because of our above data showing that PAR2 and M3 are probably tightly connected in colon primitive cells, it was important to investigate a M3-dependent regulation under PS. Incubation of colonoids from PS (Stress) males with the M3 pharmacological inhibitor 4-DAMP increased growth as well as in control conditions (Fig. 7*A*, *left*). Note that colonoids from control or PS (Stress) males were not impacted by atropine treatment (Fig. 7*A*, *right*) confirming that, among muscarinic receptors, M3 exerts a specific role in the crypt. M3 (*Chrm3*) and M1 (*Chrm1*) are the most abundant muscarinic acetylcholine receptors in the crypts (15) and the gene expression was unchanged under PS (Stress; Fig. 7*B*). Also, we measured the gene expression of cholinesterases (*Ache*, *Buche*) and the endocrine marker *Prox1* that are all implicated in the epithelial cholinergic niche (14, 15), as well as the calcium channel *Trpv4* as a common target of M3 and PAR2 signaling (37–40). Among these genes, only *Buche* expression was decreased in PS (Stress) compared with control males (Fig. 7*B*).

These data suggest that PAR2 and M3 could share a signaling pathway leading to quiescence of colon primitive cells, such as GSK3β, in both control and PS conditions. Also, PAR2 and M3 are highly expressed in secretory progenitors of the crypt (41) where the adhesive molecule CD24 is enriched (42). We analyzed the level of GSK3β, P-GSK3β and CD24 in crypts from male mice by IF. In control mice, the expression of GSK3β and P-GSK3β was diffuse, whereas CD24 was restricted to some cells at the crypt bottom where are stem cells and progenitors (Fig. 7*C*) as previously shown (43). In PS (Stress) mice, the intensity of P-GSK3β was largely reduced and more restricted to crypt bottoms, whereas GSK3β was maintained (Fig. 7*C*). In stress conditions, CD24 expression at crypt bottoms was increased compared with the control and located in the same areas than P-GSK3β (Fig. 7*C*). We further analyzed P-GSK3β and CD24 expression in colonoids (in vitro cultured crypt bottoms from control and PS mice) containing colon primitive cells (stem cells and progenitors). By contrast with crypts in situ, P-GSK3β was very low and thus in its activated form in control colonoids, and CD24 remained poorly expressed as in situ (Fig. 7*D*). M3 inhibition by DAMP had no effect on P-GSK3β or CD24 in control organoids (Fig. 7*D*). In PS (Stress) conditions, IF quantification shows that P-GSK3β remained low and that CD24 was decreased, compared with control colonoids (Fig. 7*D*). On DAMP treatment, P-GSK3β was unchanged, whereas CD24 was increased in stress colonoids (Fig. 7*D*). Altogether these data show that on stress (PS or crypt detachment from its microenvironment), GSK3β is activated by dephosphorylation and M3 may not play a critical role in that activation. On the other hand, on PS, CD24^+^ primitive cells are decreased under the control of M3 in colonoids from males whereas increased CD24 labeling colocalized with P-GSK3β at crypt bottoms in situ. As M3 has been shown to play an inhibitory role in the transition between stem cells and progenitors for intestinal differentiation (14), our results suggest that in situ some factors counteract the M3 effects.

**Figure 8.**
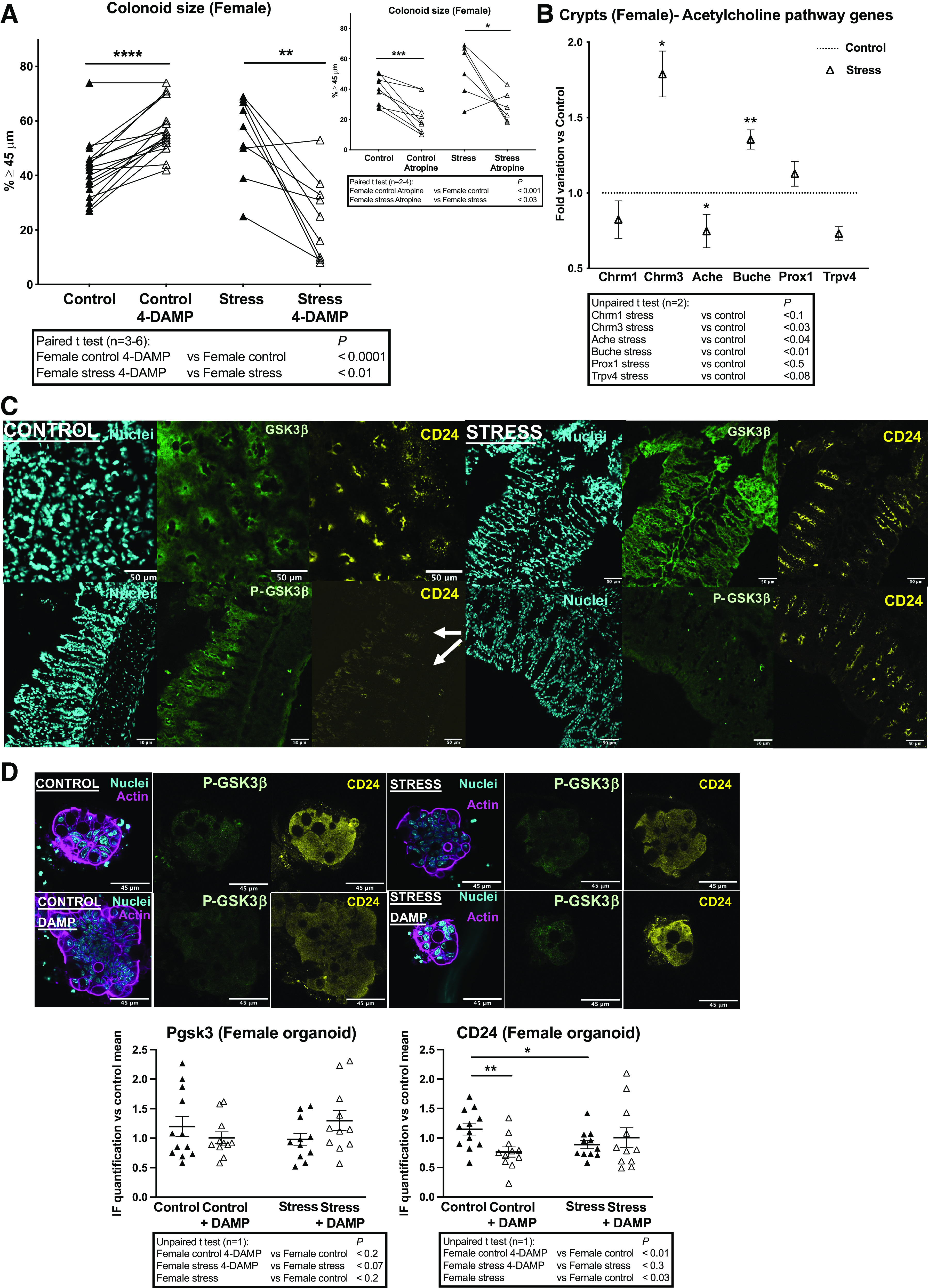
Impact of PS on M3-dependent regulation of colon primitive cells from female mice. Colon crypts were isolated from female mice whose mothers were stressed (Stress) or not (Control) during late gestation. Then colon crypts were used for gene expression analysis or cultured as colonoids. *A*: the M3-specific inhibitor 4-DAMP (10 µM) or the general muscarinic receptor inhibitor atropine (10 µM) was added to the colonoid culture as described in Fig. 1. Colonoid growth was evaluated at *day 6* of culture through diameter measurement. Around 20 colonoids were measured by assay and percentage of colonoids above the 45-µm threshold (crypt diameter) is represented. Lines between individual values show variations secondary to the treatment by 4-DAMP or atropine. Data are from 2 to 6 independent experiments (*n*) with 1–3 animals per condition. *B*: RT-qPCR was performed on mRNA isolated from colon crypts. Selected genes implicated in cholinergic regulation of colon primitive cells are shown. Data (2 independent experiments with 6–8 animals per condition) are represented as fold variation vs. Control. *C*: in situ immunolabeling of total GSK3β (green), inhibited form P-ser9 GSK3β (P-GSK3β, green) and CD24 (yellow), was realized on colon samples from female mice. Representative confocal photomicrographs of colonic tissue slices from 3 Control and 3 Stress (PS) mice (*n* = 1 experiment) are shown. Nuclei labeling (cyan) and isotype control (for CD24 antibody) labeling are shown. Scale bar = 50 µm. *D*: colonoids from Control and PS (Stress) females were treated or not with the M3 specific inhibitor 4-DAMP (10 µM) as above and immunolabeling of P-ser9 GSK3β (P-GSK3β, inhibited form) and CD24 was realized. IF was quantified from 2 to 8 colonoids per assay (1 experiment with 3 animals per condition is represented) using ImageJ software. Representation of data is vs. control mean of fluorescence as means ± SE. Representative images of immunolabeled GSK3β and CD24 in colonoids: actin (magenta) and nuclei (cyan) labeling are shown. Scale bar = 45 µm. Statistical *t* tests are shown: *P < 0.05; **P < 0.01; ***P < 0.001; ****P < 0.0001. IF, immunofluorescence; PS, prenatal stress.

The same analyses were performed in females. As in males, M3 inhibition by DAMP induced an increase of control colonoid size in females (Fig. 8*A*, *left*). Strikingly, under PS conditions, M3 inhibition induced a decrease of colonoid size in females, oppositely to males (Fig. 8*A*, *left*). This indicates that M3 has an opposite role on colonoid growth in basal or PS conditions in females. To evaluate whether the PS-triggered switch in M3-dependent regulation in females was also effective for other muscarinic receptors in the crypt, we used the global inhibitor atropine on colonoids. As shown in Fig. 8*A* (*right*), atropine induced a decrease of colonoid size in control females, which was maintained under PS (Stress). Altogether these data confirm that, among muscarinic receptors, M3 exerts a specific role in the crypt at the basal condition which is switched in PS females.

Genes implicated in the acetylcholine pathway (*Chrm1*, *Chrm3*, *Ache*, *Buche*, *Prox1*, *Trpv4*) were not differentially expressed in females compared with males (Supplemental Fig. S1*B*, *top*). Under PS (Stress), by contrast with males, *Buche* was increased in females and in parallel, an increase of *Chrm3* and a decrease of *Ache* were measured in PS females (Fig. 8*B*). As a result, crypts from PS females displayed higher *Chrm3* and *Buche* compared with PS males (Supplemental Fig. S1*B*, *bottom*).

In control female mice, the expression of GSK3β and P-GSK3β was diffuse, whereas CD24 was found more frequently at the crypt bottoms compared with males (Fig. 8*C*). In PS (Stress) female mice, the intensity of P-GSK3β was largely reduced whereas GSK3β was maintained (Fig. 8*C*). In stress conditions, CD24 expression was increased and extended in the crypts (Fig. 8*C*). In colonoids, P-GSK3β was low and similar in both control and PS (Stress) conditions, and not modified by DAMP-induced M3 inhibition in both conditions (Fig. 8*D*). CD24 was found highly expressed in control colonoids and decreased on DAMP treatment (Fig. 8*D*). In PS (Stress) conditions, CD24 was decreased compared with control and not modified by DAMP (Fig. 8*D*). Again, these data obtained with females show that, as in males, on PS or crypt detachment from its microenvironment GSK3β is activated by dephosphorylation and M3 may not play a critical role in that activation. Under PS (Stress), as in males, CD24^+^ primitive cells from females are decreased in colonoids, whereas CD24 labeling is increased in crypts in situ. Oppositely to males, in females M3 modulates CD24 expression in control but not in PS conditions. This confirms the key role of M3 in the negative control of both proliferation and differentiation of primitive cells from females that is switched off under PS.

Altogether, these results show that PS induces critical and sexually dimorphic changes in the cholinergic regulation of the crypt. In particular, colon primitive cells from females display a high susceptibility to the epithelial cholinergic niche, with M3 as a hub gene to cope with stress and to maintain growth and differentiation.

## DISCUSSION

This work aimed to highlight potential changes in ISC regulation after PS. We showed unambiguously that ISC from PS progeny copes with cell culture stress through sexually dimorphic responses. Here, the stress is triggered by de-adhesion of epithelial crypts from their colonic microenvironment in vivo, a situation that can be associated with pathological injury (44, 45). Then, in a new in vitro environment, ISC and progenitors have to survive, migrate, and proliferate to restitute the architecture and growth of the crypt, as colonoids.

The G protein-coupled receptors PAR2 and M3 play a critical role in the control of ISC and progenitor growth (9, 14, 15, 21) and are highly expressed in secretory progenitors of the crypt (41). Our data obtained in colonoids from control mice suggest that both receptors could share signaling pathways in colon primitive cells leading to quiescence, however, with a prominent role of PAR2 in males and of M3 in females. Whereas PAR2 and M3 could share Ca^2+^-dependent responses (16, 46), common pathways conducting to cell quiescence remain to be determined. GSK3β is activated in colonoids and activated downstream of PAR2 (9). As a key negative regulator of the proliferative β-catenin pathway (24), it could be implicated in PAR2- and M3-regulated quiescence. Our present data obtained with pharmacological inhibitors of PAR2 (GB83) or M3 (DAMP) were not conclusive for a PAR2- or M3-dependent regulation of GSK3β in males. This could be due to compensatory mechanisms between PAR2 and M3 that did not occur in our previous experiments with PAR2KO (9).

Under PS, the proliferation of colon primitive cells in colonoids from males is decreased through the control of PAR2 and M3 as well as the active status of GSK3β and associated with a decrease in crypts of the proliferative transcription factors *Sox9* and *Snai1*. By contrast, the proliferation of colon primitive cells from PS females is maintained as well as the proliferative role of PAR2 (21) despite an activation of GSK3β and is associated with an increase of the ligand *Wnt5a*. Sox9, Snai1, and Wnt5a play important roles in the crypt regeneration following stress (29, 30, 36, 47) and their variations could explain results of colonoid growth. Notably *Wnt5a* is regulated downstream of acetylcholine receptors (48). Furthermore, in PS females, the negative role of M3 in cell proliferation is switched to promote proliferation as well as other muscarinic receptors. This is associated with a *M3*/*Buche* expression increased and *Ache* expression decreased. Conversely, the expression of *Buche* was decreased in PS males. Thus, under PS, the PAR2 and M3 negative control of growth in the crypt is reinforced in male progeny, whereas in females, changes in the acetylcholine pathway involving M3 sustain proliferation as well as PAR2.

In both sexes, our data show that under PS there is a defect in secretory differentiation of colon primitive cells, associated with an activation of GSK3β that plays an important role in the control of stem cell differentiation (24). In PS males and females, the marker *Chga* of enteroendocrine cells is strongly decreased and active GSK3β has been implicated in the apoptosis of this cell type (49). Active GSK3β has also been shown to negatively regulate the differentiation of goblet cells (25) corroborating our data in PS males where *Muc2* marker is decreased. However, the expression of *Muc2* is maintained in PS females despite active GSK3β. Because of the negative role of M3 in goblet cell differentiation (14), a maintenance of *Muc2* may be related to the functional switch of M3 in PS female crypts. Also, in PS female crypts, the decrease of the bipotential secretory progenitor marker *Ngn3* could have improved the goblet cell differentiation at the expense of enteroendocrine cell lineage. Both M3 and PAR2 are highly expressed in secretory progenitors of the crypt (41) where the adhesive molecule CD24 is enriched (42). We measured a decreased CD24 level in both male and female PS colonoids, confirming a defect in the secretory pathway. Compared with control conditions, the M3-dependent negative regulation of CD24 secretory progenitors was lost in colonoids from PS females, whereas it was enhanced in PS males. Thus, under PS, the M3 negative control of goblet cell differentiation in the crypt is reinforced in male progeny, whereas in females M3-dependent changes in the acetylcholine pathway sustain goblet cell differentiation.

However, CD24 labeling in crypts in situ showed that CD24 is enhanced at crypt bottoms from both male and female PS mice, although CD24 remains higher in females than in males as shown in control conditions. This suggests that the inhibition of secretory differentiation from colon primitive cells measured in colonoids from PS mice could be modulated by microenvironmental factors. Both PAR2 and M3 have been implicated in the negative control of the extracellular signal-regulated kinase (ERK) related to adhesive functions of intestinal stem cells/progenitors (9, 50). Importantly, epigenetic regulation is critical to balance ERK between a proliferative role in secretory progenitors and a role in goblet cell differentiation (51). It is possible that epigenetic factors could modulate signals targeted by M3 or PAR2 such as the stress epicenter DUSP6 (9, 33), an ERK-phosphatase with a negative role on goblet cell differentiation (26), which is increased in PS females and associated with the maintenance of *Muc2* expression.

Altogether our results show that PS reinforces the capacities of PAR2 and M3 to negatively control growth and differentiation of colon primitive cells from male progeny, increasing resistance to additional stress. Colon primitive cells from female progeny acquire the capacity to activate GSK3β, a specific way downstream of PAR2 activation to survive and resist to stress such as anoikis that we previously attributed to male colon progenitors (21). The activation of GSK3β in colon primitive cells from PS females is associated with changes in the expression of potential targets of PAR2 including *Gna15* and *Cux1*. Our data show that epithelial serine protease(s) play an important role in GSK3β activation observed in PS females. Indeed, several studies have demonstrated that intestinal epithelial cells are major producers of serine proteases under physiological conditions (52, 53), as well as during pathological context such as IBS (53), or IBD (54–56). Because of modified abilities of PAR2 and M3 in the control of survival, growth, and differentiation, colon primitive cells from PS female mice should present a high potent phenotype with strong survival, proliferative/differentiation capacities, to cope with stress.

In a pathophysiological context of PS, an increased capacity to activate GSK3β in colon primitive cells of both sexes could be deleterious in case of repeated stresses. Beside inflammation, PS males should present a higher risk of defect in crypt regeneration such as observed in IBD, and PS females should present a higher risk to deregulate crypt regeneration. The phenotype of PS female primitive cells with active GSK3β and Wnt5a-dependent proliferative capacities is reminiscent of cancer stem cells (57). Therefore, this phenotype could represent a switch signature in stem cell identity (58) leading to distinct intestinal pathologies. This pathophysiological hypothesis requires further investigation. Also, it remains to determine the exact mechanism driving PS, through the identification of the molecules involved (hormones, etc.), their origin (microbiota, mother, etc.) and the molecular consequences (epigenetic modifications, etc.) that could contribute to an intestinal epithelial defect, and importantly which prenatal/postnatal periods are crucial so that these events occur.

## SUPPLEMENTAL DATA

10.6084/m9.figshare.21202118 Supplemental Fig. S1: https://doi.org/10.6084/m9.figshare.21202118.

## GRANTS

Financial support from the Agence Nationale de la Recherche (ANR; Parcure program to N.V.), from the region Occitanie, from the Bettencourt-Schueller Foundation (Coup d’élan to the organoid core facility, to N.V.), from the Fondation pour la Recherche Médicale (FRM, to N.V.), and from Région Occitanie Pyrénées-Méditerranée (to N.V.) have contributed to the completion of this research program.

## DISCLOSURES

No conflicts of interest, financial or otherwise, are declared by the authors.

## AUTHOR CONTRIBUTIONS

C.R-S. and N.C. conceived and designed research; M.B., L.G., D.S., G.P., C.R., N.C., and C.R-S. performed experiments; M.B., L.G., D.S., G.P., C.R., N.C., and C.R-S. analyzed data; M.B. and C.R-S. interpreted results of experiments; M.B. and C.R-S. prepared figures; C.R-S. drafted manuscript; C.R-S., A.D., N.V., C.D., and N.C. edited and revised manuscript; M.B., L.G., A.D., D.S., G.P., C.R., N.V., C.D., N.C., and C.R-S. approved final version of manuscript.
